# Progression of regional lung strain and heterogeneity in lung injury: assessing the evolution under spontaneous breathing and mechanical ventilation

**DOI:** 10.1186/s13613-020-00725-0

**Published:** 2020-08-06

**Authors:** Daniel E. Hurtado, Benjamín Erranz, Felipe Lillo, Mauricio Sarabia-Vallejos, Pablo Iturrieta, Felipe Morales, Katherine Blaha, Tania Medina, Franco Diaz, Pablo Cruces

**Affiliations:** 1 Department of Structural and Geotechnical Engineering, School of Engineering Pontificia, Universidad Católica de Chile, Santiago, Chile; 2 Institute for Biological and Medical Engineering, Schools of Engineering, Medicine and Biological Sciences, Pontificia Universidad Católica de Chile, Santiago, Chile; 3 Millennium Nucleus for Cardiovascular Magnetic Resonance, Santiago, Chile; 4 Centro de Medicina Regenerativa, Facultad de Medicina Clínica Alemana, Universidad del Desarrollo, Santiago, Chile; 5 Centro de Investigación de Medicina Veterinaria, Facultad de Ciencias de la Vida, Universidad Andres Bello, Santiago, Chile; 6 Pediatric Critical Care Unit, Clínica Alemana de Santiago, Santiago, Chile; 7 Pediatric Intensive Care Unit, Hospital El Carmen de Maipú, Santiago, Chile

**Keywords:** Acute lung injury, Spontaneous breathing, Mechanical ventilation, Lung strain, Lung heterogeneity, Image-based biomechanical analysis

## Abstract

**Background:**

Protective mechanical ventilation (MV) aims at limiting global lung deformation and has been associated with better clinical outcomes in acute respiratory distress syndrome (ARDS) patients. In ARDS lungs without MV support, the mechanisms and evolution of lung tissue deformation remain understudied. In this work, we quantify the progression and heterogeneity of regional strain in injured lungs under spontaneous breathing and under MV.

**Methods:**

Lung injury was induced by lung lavage in murine subjects, followed by 3 h of spontaneous breathing (SB-group) or 3 h of low *V*_t_ mechanical ventilation (MV-group). Micro-CT images were acquired in all subjects at the beginning and at the end of the ventilation stage following induction of lung injury. Regional strain, strain progression and strain heterogeneity were computed from image-based biomechanical analysis. Three-dimensional regional strain maps were constructed, from which a region-of-interest (ROI) analysis was performed for the regional strain, the strain progression, and the strain heterogeneity.

**Results:**

After 3 h of ventilation, regional strain levels were significantly higher in 43.7% of the ROIs in the SB-group. Significant increase in regional strain was found in 1.2% of the ROIs in the MV-group. Progression of regional strain was found in 100% of the ROIs in the SB-group, whereas the MV-group displayed strain progression in 1.2% of the ROIs. Progression in regional strain heterogeneity was found in 23.4% of the ROIs in the SB-group, while the MV-group resulted in 4.7% of the ROIs showing significant changes. Deformation progression is concurrent with an increase of non-aerated compartment in SB-group (from 13.3% ± 1.6% to 37.5% ± 3.1%), being higher in ventral regions of the lung.

**Conclusions:**

Spontaneous breathing in lung injury promotes regional strain and strain heterogeneity progression. In contrast, low *V*_t_ MV prevents regional strain and heterogeneity progression in injured lungs.

## Background

Supraphysiological levels of pulmonary tissue deformation during mechanical ventilation (MV), measured in terms of relative volume change, are associated with worse outcomes during acute respiratory failure [1–3]. Tissue deformation can be expressed as *global* strain, can be defined as the ratio of tidal volume (*V*_t_) over the end-expiratory lung volume (EELV) for the lung. Global strain has been used to determine safe thresholds of *V*_t_ to prevent secondary lung injury (i.e., ventilator-induced lung injury) [1].

However, lung damage can occur in patients under MV, even if they are ventilated within the defined global safety limits. Lung deformation heterogeneity and regional overdistention have been proposed as promotor mechanisms in these conditions. A spatial correlation between areas of increased *regional* strain and areas of tissue inflammation has been found in a high global strain model, demonstrating the potential role of regional biomechanical behaviors in the progression of lung injury [2]. Considering these findings, a better understanding of the spatiotemporal progression of regional strain and heterogeneity may be the key to avoid progression of damage to the lungs during respiratory failure [3].

Clinicians are now concerned by the impact of spontaneous breathing with or without MV during acute respiratory failure. Experimental and clinical studies have demonstrated that vigorous spontaneous breathing overlapping with MV may worsen lung injury [4–7]. However, in non-intubated subjects with lung injury and spontaneous unregulated ventilatory efforts, regional forces generated by the respiratory muscles may lead to injurious effects on a regional level and induce the progression of the lung injury, a phenomenon known as patient self-inflicted lung injury [8].

With these thoughts in mind, we designed this study of experimental lung injury to understand how regional lung inflation patterns evolved in time in spontaneous-breathing subjects and in under controlled low *V*_t_ MV. We hypothesize that regional deformation in lung injury progresses in time in spontaneous-breathing lungs, whereas it remains uniform in subjects under controlled MV.

## Methods

### Animal preparation

The study protocol was approved by the Universidad Andres Bello Bioethics Committee. Sprague-Dawley rats (sex paired) were considered for this study. The rats were maintained in humidity, light, and temperature-controlled environment inside a dedicated animal research facility. Food and water were provided ad libitum. After inhalatory induction with 2% isoflurane (Aesica Queenborough Ltd., UK), the rats were anesthetized by an intraperitoneal injection of ketamine (30 mg^−1^ kg^−1^, Drag Pharma Invetec S.A.) plus xylazine (5 mg^−1^ kg^−1^, Alfasan, Woerden-Holland). After tracheal instillation with 1% lidocaine (Drag Pharma, Santiago, Chile), tracheal intubation was performed with a 16G BD Angiocath^®^ catheter (Becton-Dickinson Infusion Therapy Systems Inc., Utah, USA). For intubation, an adequate level of anesthesia was assumed if the pedal reflex was absent. Otherwise, a second ketamine (15 mg^−1^ kg^−1^) plus xylazine (5 mg^−1^ kg^−1^) dose was administered.

### Lung injury model and experimental groups

Lung injury was induced by saline lavage, as previously reported [9], but we adapted the model to maintain a group without MV during the observation period after lung injury. In short, each animal was placed in supine decubitus after intubation, and one instillation of 7.5 ml^−1^ kg^−1^ of warm normal saline was flushed in the airway. The residual fluid was suctioned from the airway (surfactant depletion). After lavage, the animals were stabilized for an average time of 10 min with a volume-controlled MV strategy, using a *V*_t_ of 6 ml kg^−1^, positive end-expiratory pressure (PEEP) of 5 cmH_2_O, I:E ratio 1:2, respiratory rate (RR) of 90 breaths per minute and an inspiratory fraction of oxygen (FIO_2_) of 1, which was delivered by a VentElite^®^ Small Animal Ventilator (Holliston, MA, USA).

Block randomization was used to assign the animals to the spontaneous breathing group (SB-group) and the low *V*_t_ controlled MV group (MV-group) after induction of lung injury and stabilization. Extubation in the SB-group was performed after stabilization when spontaneous respiratory effort was detected during a gradual lowering of respiratory frequency; then, animals were supported with a FIO_2_ of 1 in the oxygen chamber (SomnoSuite^®^ for rats, Kent Scientific, Torrington, CT, USA). For the MV-group, MV was performed using the following parameters: *V*_t_ 6 ml kg^−1^, PEEP 5 cmH_2_O, I:E ratio 1:2, RR of 90 breaths per minute and FIO_2_. All subjects were placed in a prone position for the rest of the study. Rectal temperature, electrocardiogram, RR, and oxygen saturation (SpO2) were monitored and recorded using the Small Animal Physiological Monitoring System (Holliston, MA, USA). Body temperature was maintained at 38 ± 1 °C through the controlled heating surface of the system. After 15 min of clinical stability in both groups, the vital signs were registered, and the first set of images was acquired. Then, the subjects were observed and monitored for 3 h. After this period, a second set of images was obtained. During the observation phase, dissociative anesthesia was adjusted to suppress motor activity in the SB-group and to suppress the respiratory effort and ventilatory asynchrony in the MV-group. Initially, the SB-group consisted of nine subjects, and the MV-group consisted of six subjects.

At the end of the study, the animals were killed by intravenous administration of a lethal dose of thiopental (50 mg/kg, Richmond Laboratories, Buenos Aires, Argentina). A schematic depicting the experimental protocol can be found in Additional file 1: Figure S1.

### Micro-computed tomography imaging

Micro-computed tomography (micro-CT) images were acquired using a commercial SkyScan 1278 in vivo micro-CT scanner (Bruker microCT, Kontich, Belgium). Images at end-of-expiration (EE) and end-of-inspiration (EI) were acquired at the beginning (T1) and the end (T3) of the ventilation stage. The scanner includes a physiological monitoring system to track the breathing of the subject in order to deliver time-resolved four-dimensional (4D) image sequences. In the SB-group, respiratory gating based on the thorax movement was employed to reduce the effect of motion artifacts [10], while in the MV-group, the acquisition was controlled by the mechanical ventilator cycles. Scans were performed using a source voltage of 70 kV and a source current of 140 μA. The isotropic voxel resolution was 100 μm. The retrospectively synchronized “listmode” scan was performed with an exposure time of 40 ms, a scan rotation of 360° and a step of 0.75°. The entire scanning procedure took approximately 16 min. Micro-CT images were then postprocessed using the software provided by Bruker (NRecon, Tsort, DataViewer, and CTan) to increase the signal-to-noise ratio and to enhance the contrast. Ring artifact and hardening filters were employed to improve the quality of the acquired images. Median and unsharp mask filters were applied to reduce the noise and enhance the definition of boundaries in the images.

Lung images were segmented using the active-contour method implemented in the ITK-Snap software (University of Pennsylvania, Philadelphia, USA) [11]. Manual corrections were performed when necessary, and the resulting segmented images were carefully checked by independent clinical experts to assure anatomically correct structures. Two masks for EE and EI images were generated during the segmentation step. A first mask, denoted as whole-lung mask, considered the whole-lung domain. A second mask, denoted as aerated-lung mask, only considered lung regions that belonged to compartments classified as poorly aerated, normal, and hyper-aerated, according to their values of Hounsfield Units and the ranges defined for these compartments that have been reported in the literature [12–15]. Non-aerated regions were excluded from this second mask. The aerated-lung mask was then used to compute the end-of-inspiration lung volume (EILV) and end-of-expiration lung volume (EELV). Tidal volume was defined as *V*_t_ = EILV − EELV. The global strain was calculated as *V*_t_/EELV, and minute ventilation was determined as V_min_ = RR × *V*_t_.

The distribution of aeration compartments was computed from the whole-lung masked images, dividing the lung in four compartments according to the HU intensity, as described above. Aeration distribution was reported as stacked bar charts using the percentage of the total lung volume that each compartment occupied. Besides, for the SB-group, the aeration distribution of the dorsal region and of the ventral region was computed from dividing the lung image into two subregions of equal volume along the dorsal–ventral direction.

### Biomechanical analysis and regional volumetric strain maps

The image-based biomechanical analysis was performed following the approach introduced by our group in previous publications [16, 17]. In brief, the NiftyReg library [18] was employed to perform image registration between aerated-lung masks of EE and EI to obtain the displacements between the expiratory and inspiratory states of the lung. A 3D tetrahedral finite-element mesh was created from the aerated-lung mask at EI for each lung of all subjects. The displacement of the mesh from EE to EI allowed for the calculation of local volumetric strain. The biomechanical approach used in this work has been summarized in non-technical terms elsewhere [2]. Additional file 2: Figure S2 shows a schematic diagram of the sequential steps performed for obtaining the 3D regional lung strain maps, which are indicative of local parenchymal stretching [19, 20]. To allow for regional comparison between groups, lungs in each subject were divided into ten segments with approximately equal volumes along the apical–basal (AB) direction and into ten segments along the dorsal–ventral (DV) direction. By intersecting all AB and DV segments, we constructed a matrix of 10 × 10 regions of interest (ROIs) that are independent of one another. During this procedure, some AB and DV segments did not intersect, and therefore some of the ROIs were void. Weighted mean and standard deviation values of regional volumetric strain were computed for each ROI, where the sample includes tetrahedra contained in each ROI, and weighting is performed according to each tetrahedron volume. The time evolution of the regional volumetric strain at each ROI was studied by means of the regional strain progression index (SPI), defined for each ROI as SPI = (1 + ROI-mean strain at T3)/(1 + ROI-mean strain at T1). We note that SPI is a relative measure of deformation progression. An SPI = 1 implies no evolution of regional strain, an SPI > 1 is related to temporal progression (amplification) of regional strain, and SPI < 1 implies a reduction of regional strain over time. To evaluate the dispersion of regional strain in an ROI, we defined the regional strain heterogeneity index (SHI) as the coefficient of variation of the ROI strain distribution, which is expressed in terms of volumetric change, i.e., SHI = (1 + ROI standard deviation)/(1 + ROI-mean).

### Statistical analysis

A Wilcoxon signed-rank test was employed to assess intra-group differences in time for global physiologic parameters such as global strain, SpO_2_, EELV, *V*_t_, RR, and *V*_min_, as well as those parameters in the lung aeration compartments. The time progression of regional deformation in each ROI was studied by means of the Wilcoxon signed-rank test to assess absolute differences in regional strain between T1 and T3. Relative differences in SPI were studied by means of a Mann–Whitney U-test to detect if SPI was different from 1.0. The Feltz–Miller asymptotic test for the equality of coefficients of variation from k populations [21] was employed to independently detect differences of SHI between T1 and T3 for each ROI. Values are expressed as the mean ± SEM. All calculations were done using the software for statistical computing R version 3.5.3 (http://www.R-project.org/).

## Results

Surfactant depletion resulted in severe respiratory failure during the stabilization phase, with an *S*/*F* ratio 85 ± 3 mmHg for all subjects. Statistical and image analysis was carried out using five subjects in the SB-group and five subjects in the MV-group due to the following considerations: (i) mortality in the SB-group was high, with three out of nine subjects dying before completing the observation period and image acquisition; and (ii) the CT images acquired in one animal of each group displayed a notorious alteration of the thoracic–abdominal region, preventing a reliable analysis. The group weights were 271 ± 7 g and 303 ± 15 g for the SB-group and the MV-group, respectively.

Table 1 reports the physiologic parameters (SpO_2_, RR, *V*_t_, *V*_min_, EELV) and global strain for both groups under study. Additional file 3: Table S1 shows the individual physiologic data for both groups. No significant changes were detected between T1 to T3 in any of the groups.

**Table 1:** Physiologic data for the experimental groups at the beginning and at the end of the ventilation stage, either for SB-group or MV-group

Group	Time	SpO_2_ (%)	RR (1/min)	*V*_t_ (ml/kg)	*V*_min_ (ml/min*kg)	EELV (ml/kg)	Global strain (%)
SB-group (*N* = 5)	T1	87 ± 1	117 ± 10	5.4 ± 0.9	636 ± 129	26.2 ± 2.5	22.6 ± 5.6
	T3	91 ± 2	129 ± 13	6.8 ± 1.4	892 ± 224	21.5 ± 0.7	32.4 ± 7.2
MV-group (*N* = 5)	T1	92 ± 2	90 ± 0	5.6 ± 0.3	508 ± 23	33.2 ± 3.8	17.8 ± 1.9
	T3	89 ± 1	90 ± 0	5.8 ± 0.3	524 ± 24	31.2 ± 5.0	20.9 ± 3.7

EELV, *V*_min_, *V*_t_, and global strain were obtained from image analysis of μ-CT images

No significant changes were detected between T1 to T3 in any of the groups

**Fig. 1:**
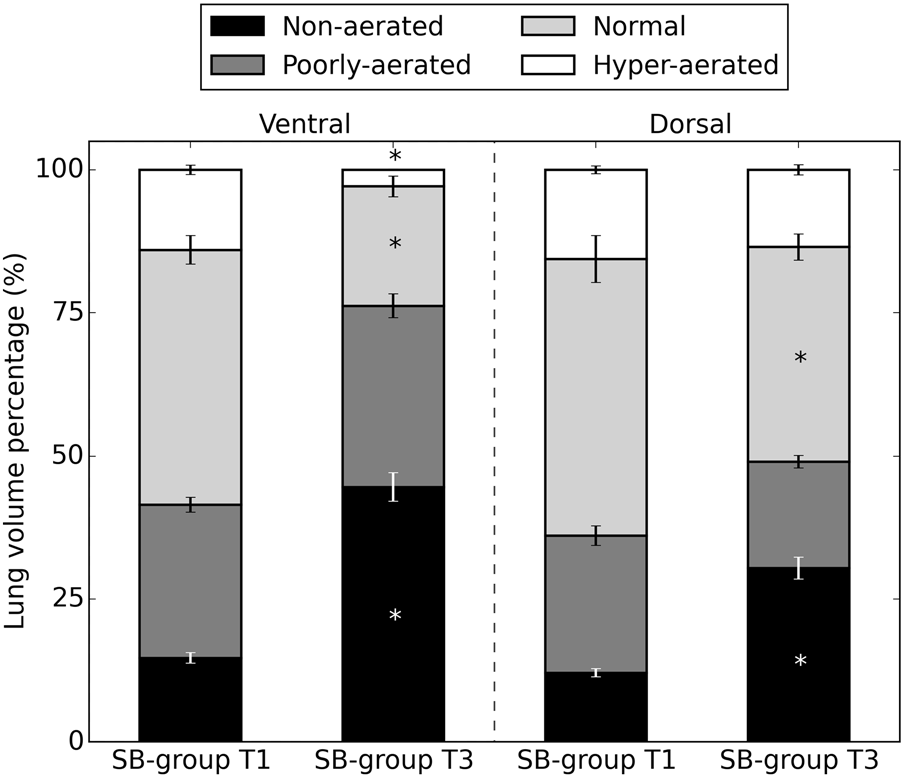
Distribution of non-aerated, poorly aerated, normal aerated and hyper-aerated lung, as defined by micro-CT scans of the total lung at end-of-expiration lung volume for SB-group and MV-group at times T1 and T3

The distribution of lung aeration is shown in Fig. 1. There were significant changes in time in lung tissue aeration in the SB-group, decreasing normal lung tissue and increasing non-aerated tissue at T3 compared to T1. There were no significant differences in MV-group. Additional file 4: Figure S3 shows the dorsal and ventral distributions of aeration compartments in the SB-group at T1 and T3. When analyzing the changes in aeration in the SB-group, we found an increase of 29.9% in the non-aerated ventral region and an increase of 17.7% in the non-aerated dorsal region. Additional file 5: Figure S4 shows CT images at EE and EI for T1 and T3 in SB-group. At EE, we observe the collapse progression over time, particularly at the ventral regions. At EI, we observe a progression of aeration in the basal-dorsal region.

**Fig. 2:**
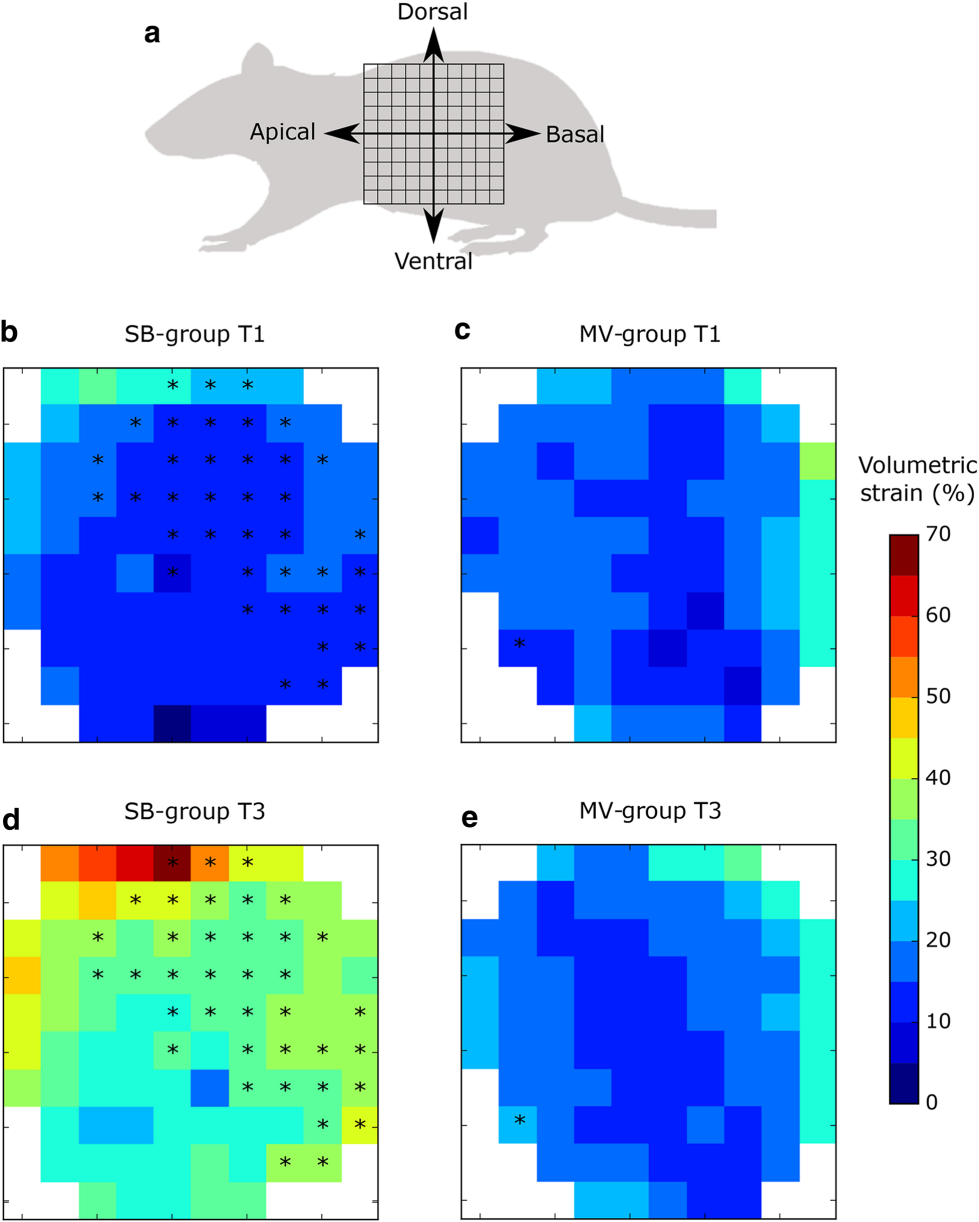
ROI array maps of the regional volumetric strain. **a** Schematic showing the apical–basal and ventral–dorsal directions of array maps. **b** Regional strain in the SB-group at T1. **c** Regional strain in the MV-group at T1. **d** Regional strain in the SB-group at T3. **e** Regional strain in the MV-group at T3. Significant within-subject differences are denoted by * (*p* < 0.05)

ROI array maps reporting ROI-mean regional volumetric strain for the SB-group and MV-group at T1 and T3 are shown in Fig. 2 (see Fig. 2a for a sketch depicting the apical–basal and ventral–dorsal directions). When comparing ROIs in T1 and T3, there was a significant increase in regional volumetric strain in 38 out of 87 ROIs (43.7%), which were predominantly located in the basal-dorsal quadrant (Fig. 2b, d). In contrast, in the MV-group, only 1 out of 84 ROIs (1.2%) was found to be significantly different between T1 and T3 (Fig. 2c, e). Additional file 6: Figure S5 shows the 3D regional volumetric strain maps for representative subjects of the SB-group and MV-group at T1 and T3.

**Fig. 3:**
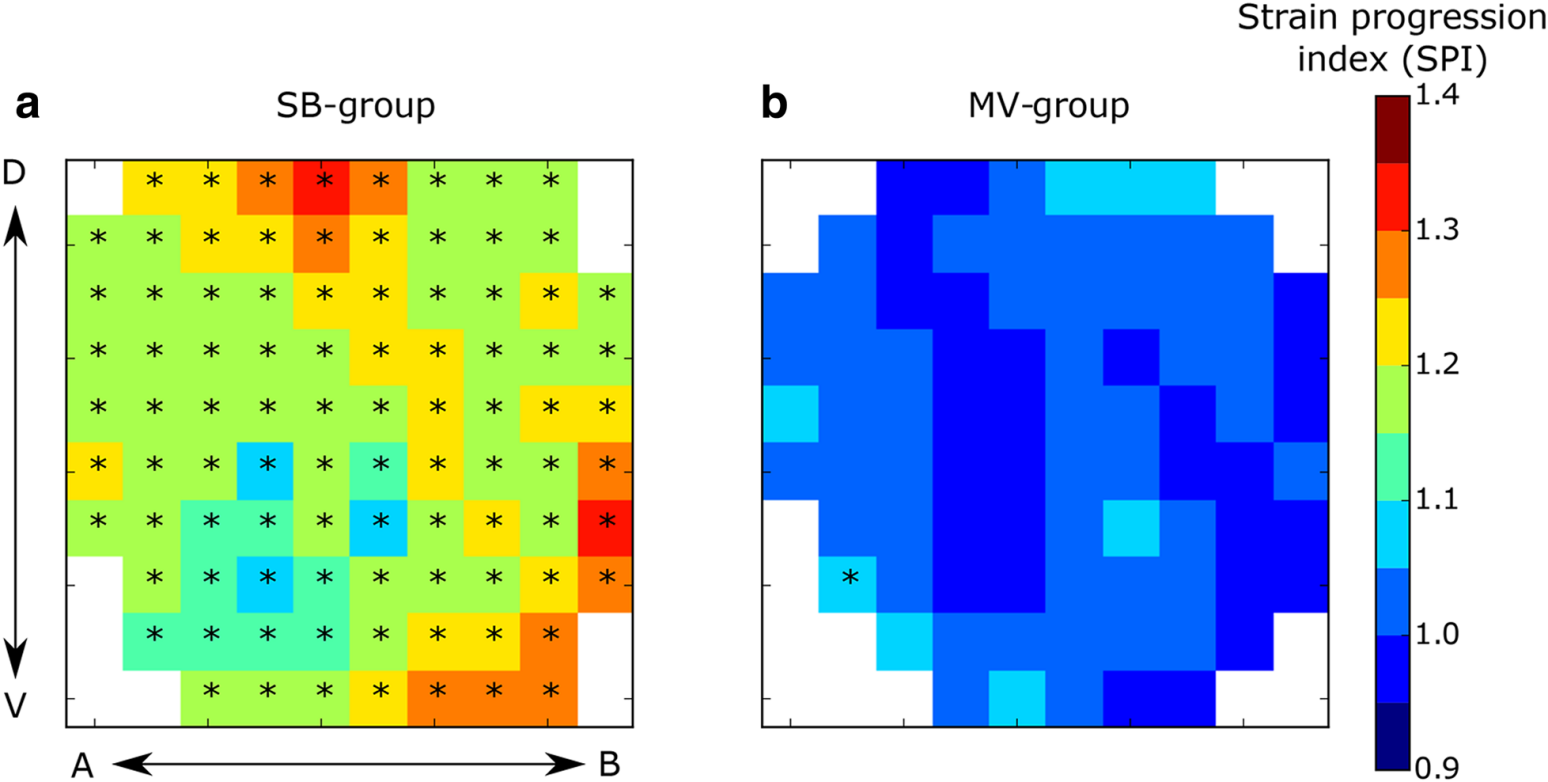
ROI array maps of the strain progression index (SPI). **a** SB-group, **b** MV-group. Significant within-subject differences are denoted by * (*p* < 0.05)

When analyzing volumetric regional strain over time, all ROIs in the SB-group (91 out of 91) had a significant SPI greater than 1, showing progression (Fig. 3a). A spatially homogeneous progression trend is therefore observed for the whole lung in this group. In contrast, in 83 out of 84 ROIs of the MV-group, SPI was not different than 1, meaning an absence of progression of volumetric strain. ROI array maps reporting the ROI-mean SPI for the SB-group and MV-group are shown in Fig. 3.

**Fig. 4:**
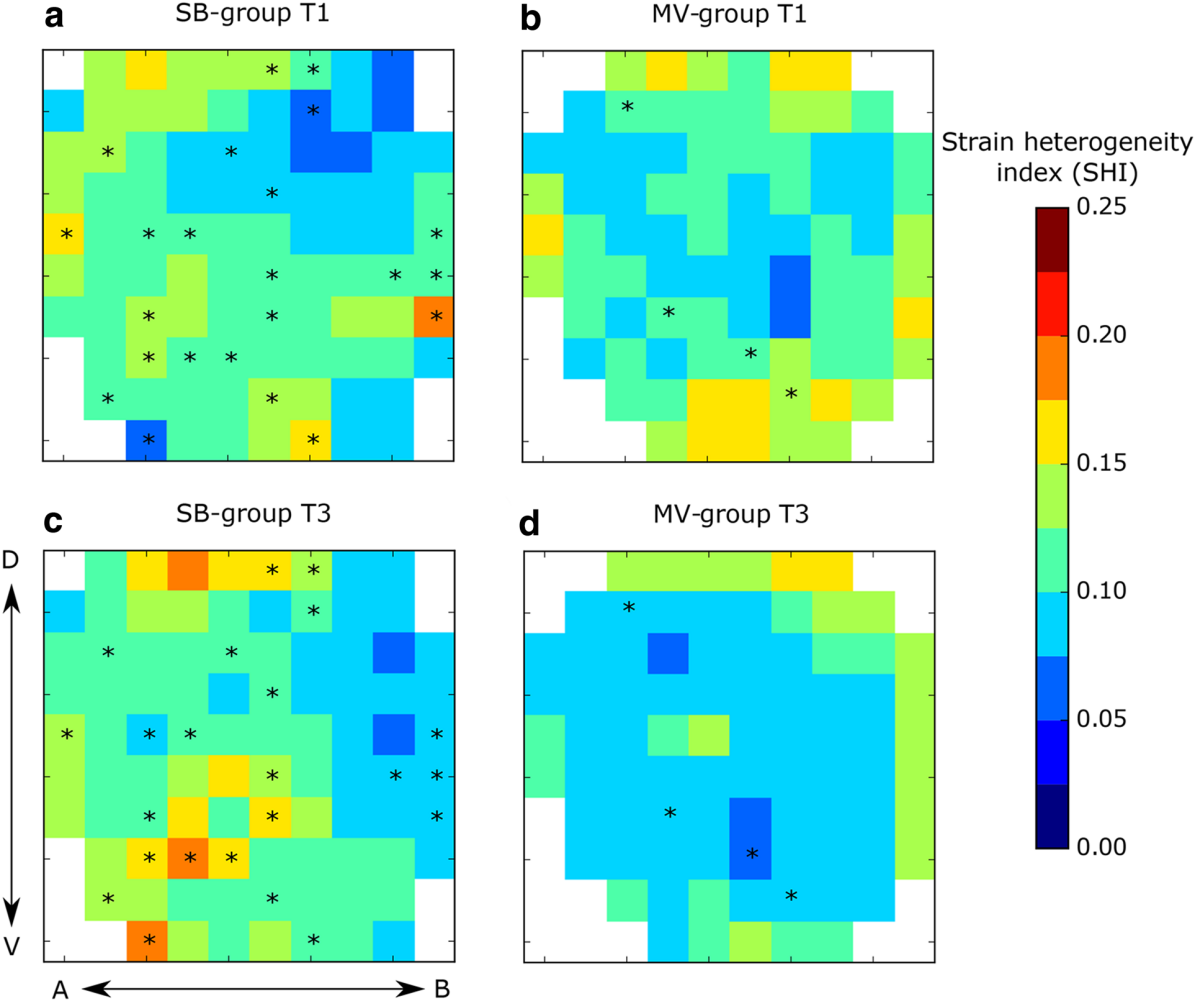
ROI array maps of the strain heterogeneity index (SHI). **a** SB-group at T1, **b** MV-group at T1, **c** SB-group at T3, **d** MV-group at T3. Significant within-subject differences are denoted by * (*p* < 0.05)

Heterogeneity of regional deformation was assessed through the SHI, for which ROI arrays are reported in Fig. 4. In the SB-group, we found a significant increase in time in SHI in 24 out of 91 ROIs (26.4%) (Fig. 4a, c). In the MV-group (Fig. 4b, d), only 4 out of 84 ROIs resulted in a significant increase of SHI between T1 and T3 (4.7%).

## Discussion

In this work, we studied the lung regional strain distribution, heterogeneity, and deformation progression in subjects spontaneously breathing and subjects on controlled low-*V*_t_ MV in a murine lung-injury model. We found that a significant progression in regional volumetric strain and heterogeneity was observed after 3 h of spontaneous breathing in injured lungs. Changes in lung regional strain during spontaneous breathing were concurrent with the tomographic progression of the nonaerated-tissue compartment of the lung and a reduction of the normal-tissue compartment, in accordance with de-recruitment phenomenon, with collapse progression being higher in ventral regions of the lung. In contrast, the MV-group had limited progression of the regional strain and heterogeneity at the end of the study.

A key finding of our study is that regional strain significantly progressed in the SB-group, but did not result in major changes in the MV-group. We note that in the SB-group, global strain increased in roughly 50% from T1 to T3, but due to intra-group variability and the small sample size, this mean increase did not result in significant differences. While this situation is a limitation of the study, it also highlights the high sensitivity of regional deformation analysis in detecting strain progression when compared to global strain. Another interesting finding is that strain progression was more substantial in the dorsal and basal regions in SB-group. This observation is supported by the fact that the progression of the lung collapse is stronger in the ventral areas than in the dorsal areas. Since collapsed tissue is not expected to deform, it is the dorsal region the one expected to deform the most, which is confirmed by our regional strain analysis. These results suggest the deformation mechanisms associated with the contraction of the diaphragm are relevant to regional deformation. The caudal movement of the diaphragm is relative to its initial relationship with costal insertion, so the capacity to generate force in the caudal direction increases proportionally to the reduction of EELV, as in severe lung injury [22]. These findings are in agreement with the experiment of Yoshida and coworkers [23], where spontaneous breathing was beneficial in subjects with lung injury under MV when its severity was mild. The opposite effects occurred when the lung injury was severe, in which spontaneous breathing amplified the injury, thus increasing transpulmonary pressures, atelectasis, cyclic collapse, and histological signs of damage.

Similar to the findings of Yoshida et al. in mechanically ventilated subjects with severe lung injury, we found more lung damage in SB-group. The paradox of spontaneous breathing and lung damage can be explained by the solid-like behavior of the injured lungs. Contraction of the diaphragm generates non-uniform fluctuations of pleural pressure across the lung surface, producing an unsuspected overstretch in dependent regions and displacement of alveolar gas to non-dependent regions of the lungs (i.e., Pendelluft) [23]. In our study, the progression of non-aerated tissue in the SB-group may have intensified these phenomena, resulting in an imperfect elastic anisotropic inflation and amplifying the damage in the poorly aerated compartment of the lungs [4]. Another finding in our study was the progression of the heterogeneity of deformation of the lung, measured in terms of regional SHI, in the SB-group. This observation suggests that sustained vigorous spontaneous ventilatory efforts might promote the progression of deformation heterogeneity in subjects with severe lung injury. In contrast, subjects on controlled MV showed fewer ROIs with progression of SHI.

Inhomogeneity of ventilation has been proposed as a promoter of lung injury associated with ventilatory support. Lung injury promoters are responsible for the amplification of damage injured lungs, even when MV parameters are within standard safety limits (non-harmful). The concept of stress raisers has been introduced to explain the amplification of damage in areas of high inhomogeneity [24, 25], linking the biological response in the lung parenchyma to the regional deformation in localized areas of the lung. The regional analysis showing inhomogeneity in the SB-group suggests that injurious patterns of ventilation in subjects without MV (spontaneous breathing), such as tidal recruitment, anisotropic inflation, and Pendelluft phenomena, among others, can be associated with progression of injury, although the method we used cannot accurately characterize them. These findings take on particular translational relevance because regional differences in tissue aeration have been related to stress raisers and in patient mortality [26]. It is important to note that regional stress is related to regional strain by means of constitutive relations (regional elastance); since regional strain can be directly estimated from image-based biomechanical analysis, it may serve as a better predictor of regional stress [27].

It has been proposed that hyperventilation, due to vigorous diaphragm contraction, can amplify the lung injury. Surprisingly, we did not find significant changes in *V*_t_ in SB-group over time. With these observations, an important question arises: if strain increases in spontaneously breathing subjects, why *V*_t_ does not change? Several mechanisms might explain this conundrum. First, high respiratory drive progressively induces higher inspiratory flow over time. In the inhomogeneous lung, as SB-group, fast alveolar units received more air, whereas the slow ones got deaerated, similar to other observations in subjects under MV [28, 29]. Also, high peak inspiratory flow in spontaneous breathing (deaccelerating pattern) increases the damage because the viscoelastic adaptation of the lung parenchyma does not have enough time to dissipate harmful forces [30]. Second, the Hering–Breuer reflex is a mechanism that can limit *V*_t_ during spontaneous breathing in subjects with a high respiratory drive. Third, vigorous breathing efforts in subjects with lung injury cannot be adjusted or regulated, even with appropriate sedation [23]. A larger amplitude of the diaphragm caudal movement generates an excessive negative intrathoracic and interstitial pressure, contributing to venous return and formation of edema [31].

Concurrent with the progression of regional strain and heterogeneity, the SB-group displayed an increase in the non-aerated compartment and a reduction of the normally aerated compartment, which is associated with alveolar collapse. This result is in agreement with observations in subjects with acute lung injury breathing spontaneously under MV [4], and the ones described by Mascheroni et al. in an experimental ovine study. The authors observed a severe deterioration of pulmonary function after 3.5–13 h of pharmacologically induced hyperventilation in spontaneously breathing animals without lung disease. MV and pseudoparalysis prevented these alterations. This study confirms that vigorous spontaneous ventilation can affect the lung, and controlled MV can prevent or attenuate the damage of the lung in this setting [32]. The development of hydrostatic lung edema in the SB-group might be a possible interpretation of these findings. In this group, a higher negative pleural pressure caused by more substantial spontaneous breathing efforts increases the transvascular alveolar pressure, which in turn results in augmented lung perfusion and finally, in edema [33–37]. Also, we need to consider that lung inflammation in regions exposed to high strain might lead to an increase in permeability; thus, they are more susceptible to edema.

Our work suffers from certain limitations that should be improved in future experiments. Operational restrictions and scanning-time demand imposed by the micro-CT scanner did not allow for the use of invasive monitoring. It took over 15 min for a full scan, and inside the scanning chamber, the spontaneous respiratory effort could not be monitored using esophageal pressure monitoring; monitoring gas exchange was also not possible. These restrictions prevented us from quantifying the parameters of global lung mechanics—oxygenation and ventilation—which are needed to classify the severity of the lung injury as well as the intensity of the respiratory effort. We note that subjects in the SB-group were also under anesthesia, which may modify the respiratory pattern. In addition, it is important to mention that our strain measurements in the MV-group are related to dynamic strain and do not account for the deformations that may occur due to the use of PEEP volume, which we believe are small compared to the dynamic strain. A technical limitation concerned with the image registration and biomechanical analysis is the fact that currently, the regional strain can only be computed in aerated regions of the lung. In particular, regional strain in the non-aerated areas was not calculated. This technical limitation does not allow us to conclude regarding the deformation of collapsed areas of the lung. Despite these limitations, we highlight the unique character of this experimental design to study “patient self-inflicted lung injury” (P-SILI). We measured the regional strain and heterogeneity in spontaneously breathing subjects in the whole lung.

Future studies should include a regional analysis of inflammation and atelectasis that could be spatially related to the different deformation measures proposed in this study to confirm correlations between regional deformation, tissue inflammation and edema, and their progression over time.

## Conclusions

Spontaneous breathing can induce progression of lung injury by many mechanisms, a phenomenon known as P-SILI. We identified a progression of regional deformation and heterogeneity in injured lungs under spontaneous breathing, but not in low *V*_t_ MV subjects. This topic has profound implications in translational research, as patients with acute respiratory insufficiency can spontaneously breathe for extended periods before starting appropriate MV support, and also during unsuccessful weaning [38, 39]. Understanding the mechanisms involved in the progression of lung damage and its main determinants—heterogeneity and stress raisers, among others—will better support the decision to start or hold off MV support, thus balancing risks and benefits and potentially improving the clinical outcome. Low *V*_t_ MV is a strategy that can attenuate stress raising phenomena, thus reducing the maldistribution of regional strain dictated by lung heterogeneity [26]. Future studies need to assess whether other modalities of respiratory support, such as noninvasive MV and high-flow nasal cannulas, can attenuate the progression of lung injury and regional volumetric lung strain.

## Supplementary information

**Additional file 1: Figure S1.** Schematic of the experimental protocol.

**Additional file 2: Figure S2.** Schematic of the image acquisition process and image-based biomechanical analysis employed to construct three-dimensional lung regional strain maps.

**Additional file 3: Table S1.** Individual physiologic data for both groups. (a) SB-group at T1, (b) SB-group at T3, (c) MV-group at T1, (d) MV-group at T3.

**Additional file 4: Figure S3.** Aeration distribution of dorsal and ventral regions in the SB-group at T1 and T3

**Additional file 5: Figure S4.** CT images of a representative subject in the SB-group showing the regional progression of lung collapse at the end of expiration (EE) and aeration at the end of inspiration (EI): (i) Subject at T1 during EE, (ii) Subject at T3 during EE, (iii) Subject at T1 during EI, (iv) Subject at T3 during EI.

**Additional file 6: Figure S5.** Regional volumetric strain maps for representative subjects of the SB-group (top row) and the MV-group (bottom row) at T1 and T3. Progression of regional strain and heterogeneity in time is observed for the SB-subject, which reaches volumetric strain levels of up to 80%. Regional strain distribution remains uniform and homogeneous in the MV subject.

## Data Availability

The datasets used and analyzed during the current study are available from the corresponding author on reasonable request.

## Abbreviations

ALI: Acute lung injury
ARDS: Acute distress respiratory syndrome
CT: Computed tomography
EELV: End-expiratory lung volume
EILV: End-of-inspiration lung volume
FIO_2_: Inspiratory fraction of oxygen
HU: Hounsfield units
MV: Mechanical ventilation
PEEP: Positive end-expiratory pressure
P-SILI: Patient self-inflicted lung injury
ROI: Regions of interest
RR: Respiratory rate
SHI: Strain heterogeneity index
SPI: Strain progression index
SpO_2_: Oxygen saturation
*V*_min_: Minute ventilation
*V*_t_: Tidal volume
