# Supplementary figures and images for: Progression of regional lung strain and heterogeneity in lung injury: assessing the evolution under spontaneous breathing and mechanical ventilation
Source: Ann Intensive Care. 2020 Aug 6;10:107. doi: 10.1186/s13613-020-00725-0 (PMC7407426; doi:10.1186/s13613-020-00725-0)

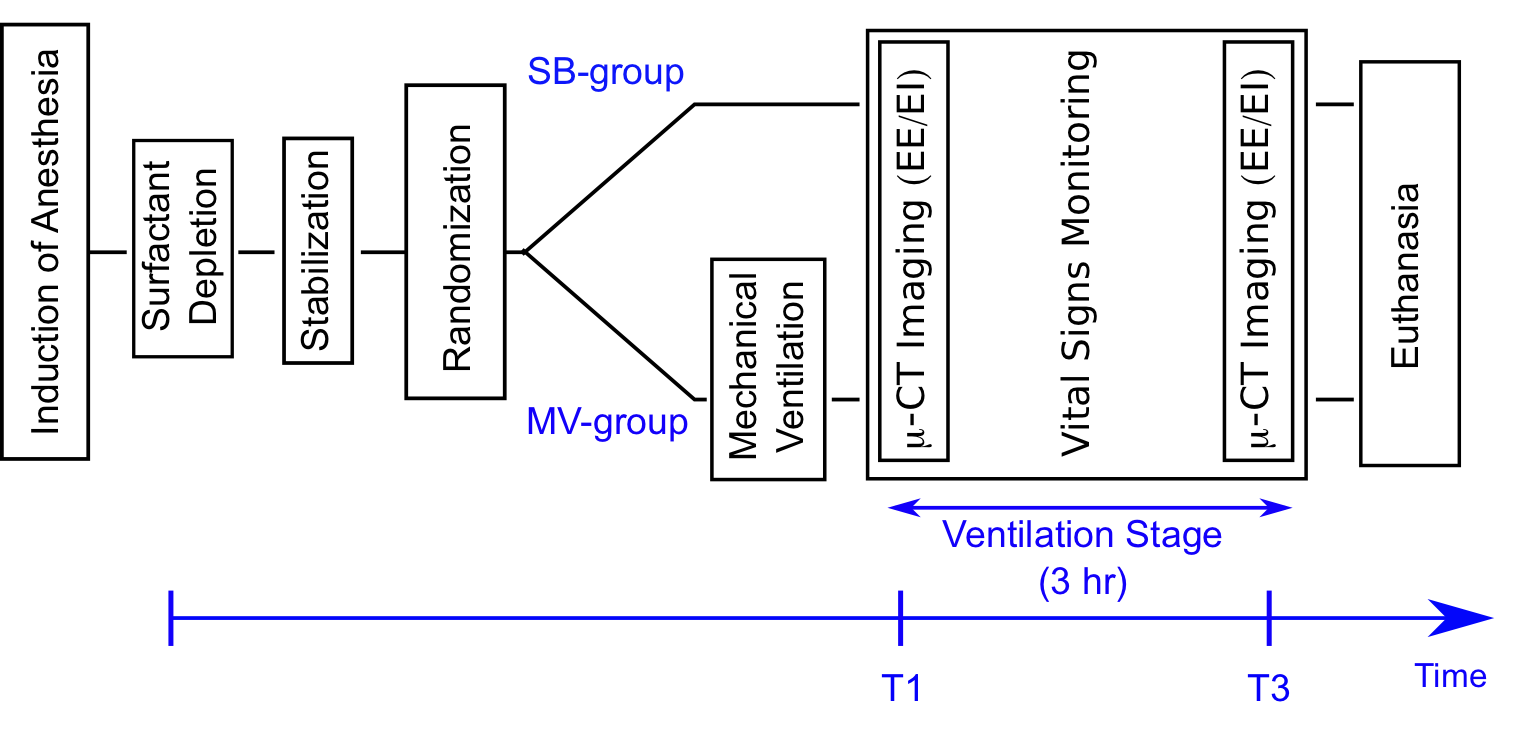

Supplement: Supplementary file 1 — Additional file 1: Figure S1. Schematic of the experimental protocol. [file 13613_2020_725_MOESM1_ESM.tiff]

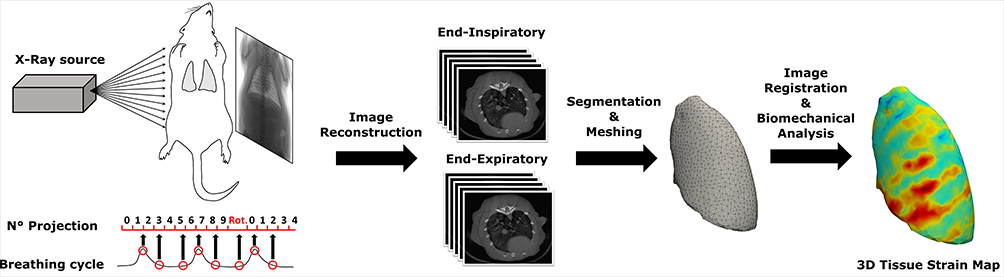

Supplement: Supplementary file 2 — Additional file 2: Figure S2. Schematic of the image acquisition process and image-based biomechanical analysis employed to construct three-dimensional lung regional strain maps. [file 13613_2020_725_MOESM2_ESM.tif]

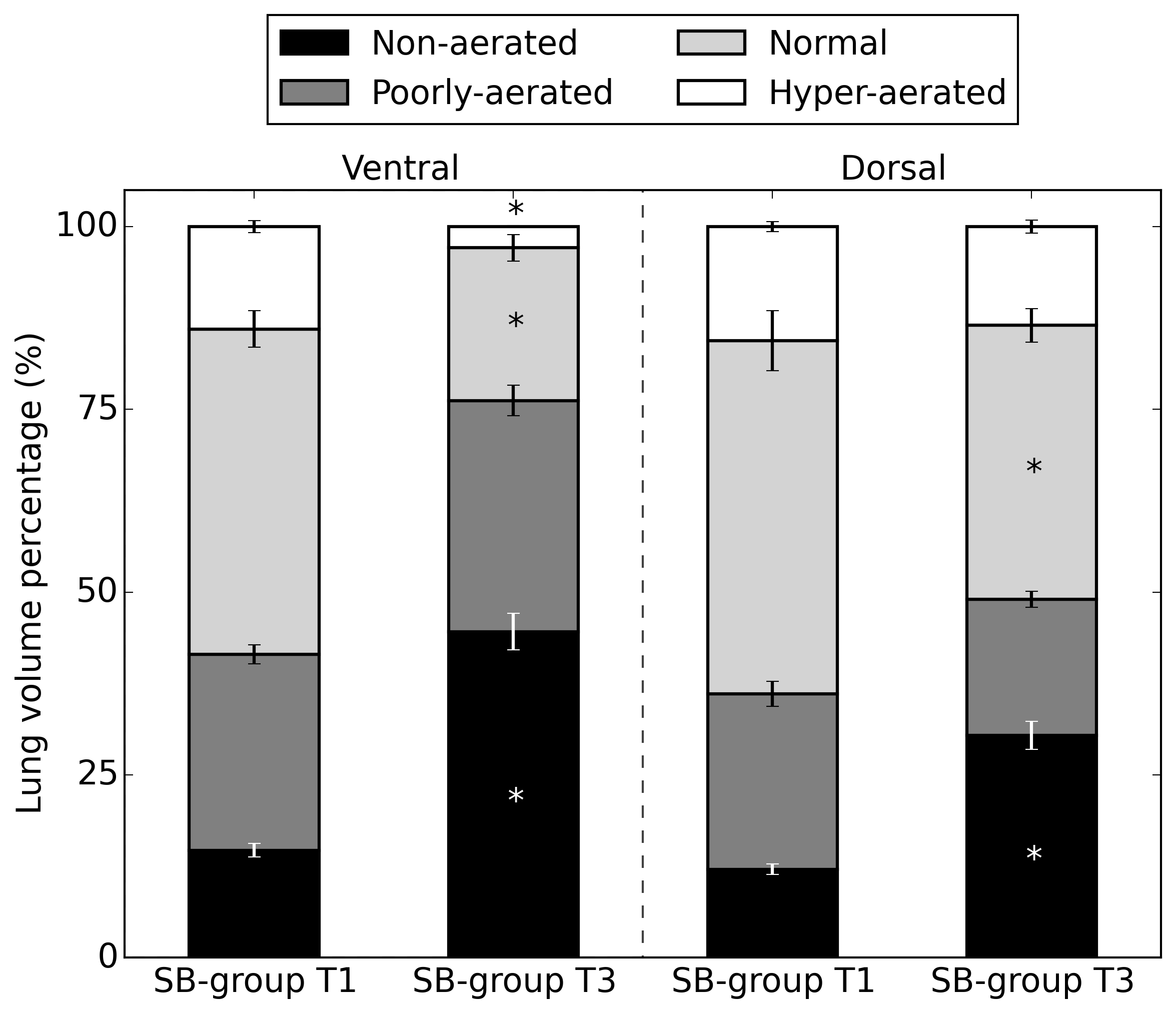

Supplement: Supplementary file 4 — Additional file 4: Figure S3. Aeration distribution of dorsal and ventral regions in the SB-group at T1 and T3 [file 13613_2020_725_MOESM4_ESM.tiff]

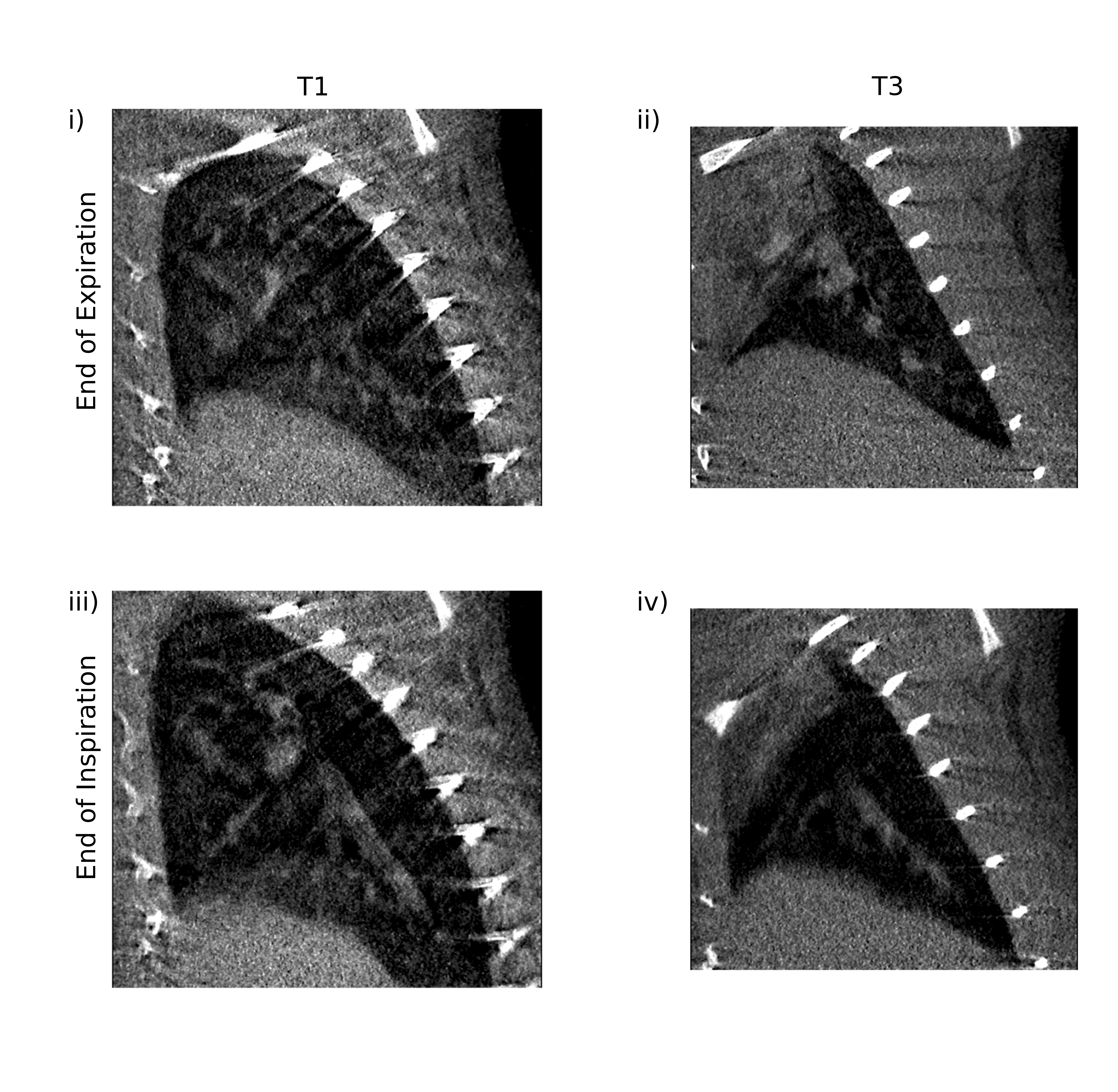

Supplement: Supplementary file 5 — Additional file 5: Figure S4. CT images of a representative subject in the SB-group showing the regional progression of lung collapse at the end of expiration (EE) and aeration at the end of inspiration (EI): (i) Subject at T1 during EE, (ii) Subject at T3 during EE, (iii) Subject at T1 during EI, (iv) Subject at T3 during EI. [file 13613_2020_725_MOESM5_ESM.tiff]

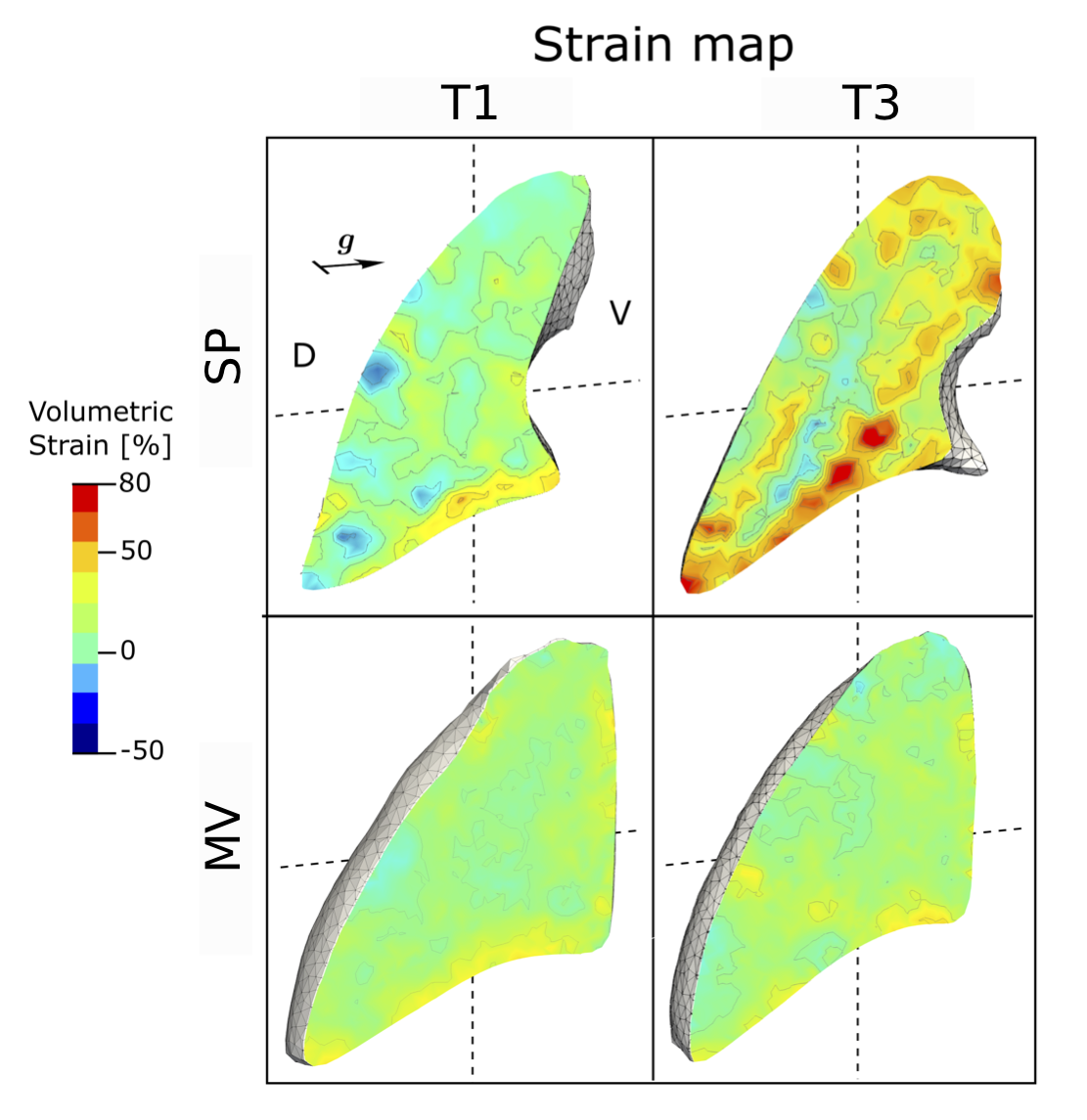

Supplement: Supplementary file 6 — Additional file 6: Figure S5. Regional volumetric strain maps for representative subjects of the SB-group (top row) and the MV-group (bottom row) at T1 and T3. Progression of regional strain and heterogeneity in time is observed for the SB-subject, which reaches volumetric strain levels of up to 80%. Regional strain distribution remains uniform and homogeneous in the MV subject. [file 13613_2020_725_MOESM6_ESM.tiff]
