# Supplementary material for: Progression of regional lung strain and heterogeneity in lung injury: assessing the evolution under spontaneous breathing and mechanical ventilation
Source: Ann Intensive Care. 2020 Aug 6;10:107. doi: 10.1186/s13613-020-00725-0 (PMC7407426; doi:10.1186/s13613-020-00725-0)
Supplement: Supplementary file 3 — Additional file 3: Table S1. Individual physiologic data for both groups. (a) SB-group at T1, (b) SB-group at T3, (c) MV-group at T1, (d) MV-group at T3. [file 13613_2020_725_MOESM3_ESM.docx]

**Table E1 (Supp Material)** Individual physiologic data for both groups. (a) SB-group at T1, (b) SB-group at T3, (c) MV-group at T1, (d) MV-group at T3. Data is expressed as mean value ± SEM. No significant changes were detected between T1 to T3 in any of the groups.

**Table E1a:** Individual physiologic data for the SB-group at T1.

| **Subject**  **ID** | **SpO_2_ (%)** | **RR (1/min)** | **VT (ml/kg)** | **V_min_ (ml/min*kg)** | **EELV (ml/kg)** | **Global strain (%)** |
| --- | --- | --- | --- | --- | --- | --- |
| 01 | 89 | 139 | 3.6 | 495 | 24.9 | 14.3 |
| 02 | 86 | 94 | 4.3 | 404 | 29.9 | 14.4 |
| 03 | 85 | 123 | 4.0 | 490 | 32.8 | 12.1 |
| 04 | 87 | 136 | 8.3 | 1123 | 25.1 | 32.9 |
| 05 | 88 | 94 | 7.1 | 667 | 18.1 | 39.2 |
| Mean ± SEM | 87 ± 1 | 117 ± 10 | 5.4 ± 0.9 | 636 ± 129 | 26.2 ± 2.5 | 22.6 ± 5.6 |

**Table E1b:** Individual physiologic data for the SB-group at T3.

| **Subject**  **ID** | **SpO_2_ (%)** | **RR (1/min)** | **VT (ml/kg)** | **V_min_ (ml/min*kg)** | **EELV (ml/kg)** | **Global strain (%)** |
| --- | --- | --- | --- | --- | --- | --- |
| 01 | 85 | 87 | 5.7 | 496 | 23.7 | 24.1 |
| 02 | 93 | 133 | 4.1 | 545 | 21.5 | 19.1 |
| 03 | 94 | 140 | 4.1 | 574 | 21.7 | 18.9 |
| 04 | 94 | 167 | 9.6 | 1603 | 19.0 | 50.5 |
| 05 | 90 | 116 | 10.7 | 1241 | 21.6 | 49.5 |
| Mean ± SEM | 91 ± 2 | 129 ± 13 | 6.8 ± 1.4 | 892 ± 224 | 21.5 ± 0.7 | 32.4 ± 7.2 |

**Table E1c:** Individual physiologic data for the MV-group at T1.

| **Subject**  **ID** | **SpO_2_ (%)** | **RR (1/min)** | **VT (ml/kg)** | **V_min_ (ml/min*kg)** | **EELV (ml/kg)** | **Global strain (%)** |
| --- | --- | --- | --- | --- | --- | --- |
| 06 | 84 | 90 | 5.4 | 486 | 35.2 | 15.3 |
| 07 | 98 | 90 | 5.5 | 495 | 36.1 | 15.2 |
| 08 | 94 | 90 | 5.8 | 522 | 31.3 | 18.5 |
| 09 | 93 | 90 | 6.5 | 585 | 43.4 | 15.0 |
| 10 | 92 | 90 | 5.0 | 450 | 20.2 | 24.8 |
| Mean ± SEM | 92 ± 2 | 90 ± 0 | 5.6 ± 0.3 | 508 ± 23 | 33.2 ± 3.8 | 17.8 ± 1.9 |

**Table E1d:** Individual physiologic data for the MV-group at T3.

| **Subject**  **ID** | **SpO_2_ (%)** | **RR (1/min)** | **VT (ml/kg)** | **V_min_ (ml/min*kg)** | **EELV (ml/kg)** | **Global strain (%)** |
| --- | --- | --- | --- | --- | --- | --- |
| 06 | 85 | 90 | 5.9 | 529 | 19.5 | 30.1 |
| 07 | 92 | 90 | 6.5 | 585 | 44.2 | 14.7 |
| 08 | 92 | 90 | 4.9 | 440 | 38.6 | 12.7 |
| 09 | 88 | 90 | 6.1 | 545 | 34.0 | 17.8 |
| 10 | 90 | 90 | 5.8 | 523 | 19.8 | 29.4 |
| Mean ± SEM | 89 ± 1 | 90 ± 0 | 5.8 ± 0.3 | 524 ± 24 | 31.2 ± 5.0 | 20.9 ± 3.7 |
